# Development and validation of Attitude Toward Nutrition Counselling Questionnaire for use among Kuwaiti healthcare professionals

**DOI:** 10.1186/s13104-020-4905-9

**Published:** 2020-02-07

**Authors:** Nouf S. Al-Mughamis, Abdullah A. Alayoub, Hafsa Meraj, Ahmed Waqas

**Affiliations:** 1 Ministry of Health of Kuwait, Kuwait City, Kuwait; 2 Kuwait Cancer Control-Ministry of Health of Kuwait Center, Shuwaikh, Kuwait; 3 Shifa College of Medicine, Islamabad, Pakistan; 4 University of Liverpool, Liverpool, UK

**Keywords:** Nutrition, Questionnaire, Scale, Knowledge, Dietician, Arabic, Attitude

## Abstract

**Objective:**

This study aims to report the developmental processes and validation of *Attitude Toward Nutrition Questionnaire* in Arabic language.

**Results:**

A total of 173 (response rate = 86.93%) participants responded to the survey. There were a total of 92 (53.2%) nutritionists and 81(46.8%) doctors/surgeons. Principal component analyses revealed followed by visualization of Cattell’s scree plot, suggested a four-factor solution for the 36-item Attitude Toward Nutrition Counselling Questionnaire. It was found to have an acceptable validity. These four factors cumulatively explained 37.9% of the variance in the factor structure of the ATNQ. Cronbach’s alpha revealed an acceptable level of reliability for each subscale of the ATNQ. The first subscale named “Factual knowledge about nutrition” comprised of nine items. It yielded a Cronbach’s alpha value of 0.78. The second subscale “knowledge about nutrition in morbidities” comprised of seven items and yielded a Cronbach’s alpha value of 0.71. The third subscale “counselling of patients” comprised of 11 items and yielded a Cronbach’s alpha of 0.68. The fourth subscale comprising nine items yielded a Cronbach’s alpha value of 0.64 and was named, “Dietary programs and supplementation”.

## Introduction

Nutrition is an essential component of medical care, bearing an association with prognosis, management and outcome of chronic diseases such as coronary and metabolic diseases [1–3]. Therefore, it is crucial for medical professionals to have an adequate knowledge of nutrition in order to improve patient outcomes [4]. Unfortunately, several investigations have shown poor knowledge of nutrition among medical professionals, partly owing to their deficient education during preclinical and clinical training [5–7]. It is, therefore, imperative that nutrition knowledge of practicing medical professionals be assessed to overcome gaps in knowledge and improve patient care [8].

It is important to develop tools and assessment questionnaires pertaining to attitude, knowledge and practices regarding nutrition. However, no such tool has been developed for the middle eastern population of Kuwait. Although an earlier study assessed nutrition knowledge of physicians in Kuwait, a psychometrically valid nutrition knowledge questionnaire has not yet been developed [8]. The present study, therefore, addresses this paucity of data. This study aims to report the developmental processes and validation of Attitude Toward Nutrition Counselling Questionnaire (ATNQ).

## Main text

### Methods

#### Questionnaire development

The development of the questionnaire was done in a multiphasic process. In the first phase, a thorough review of the literature was conducted to identify the questionnaires and modalities that have been used in the Middle East [9–16]. The items in these questionnaires were then checked for their suitability and adaptation by an experienced dietician and a public health researcher (NS & AW) inclusion in the *ATNQ*. In addition, several more items were developed, based upon the Ajzen’s theory of Planned Behaviour, which states that an action requires three pre-meditated components; attitudes, knowledge and practice [17]. Overall, these items assessed the participants on attitudes, knowledge and practices related to nutrition and diet in their clinical practice. Responses on these items were assessed using a five-point Likert Scale ranging from strongly agree to strongly disagree.

#### Pilot survey

In the next phase, we recruited 18 dieticians, nutritionists (n = 6), medical students (n = 3) and medical doctors (n = 9). The participants were requested to respond to the ATNQ and then comments on its suitability, strengths and weaknesses. Using open ended questions, they were also requested to comment on the items to be excluded or rephrase sentences for an improved comprehension. They were also requested to suggest more items that could be added in the questionnaire. Typical comments raised were to mention measurement units as mmol/l instead of mg/dl; less suitability of the questionnaire for medical students and a high number of questionnaire items. It was also suggested that questions pertaining to physical activity, renal-nutrition, bariatric surgery, physical activity and knowledge acquisition behaviors be added in the questionnaire. After the pilot study, we made necessary changes in the questionnaire, yielding a total of 52 items in the finalized questionnaire (Table 1). It is important to note that the data collected from the pilot survey was not included in the final dataset.

**Table 1:** Rotated component matrix showing dimensionality of the Attitude Toward Nutrition Counselling Questionnaire

Statements	Factors			
	Factual knowledge	Morbidities	Counselling of patients	Dietary programs and supplementation
Increased intake of fruits and vegetables is associated with good blood pressure control			0.558	
A traditional Mediterranean diet focuses on a reduction in total fat intake				0.362
Research has shown strong evidence that Atkin’s low carbohydrate diet regime leads to a good cardiovascular health				0.479
In comparison with nutrition, proper exercise is more important in reduction of cardiovascular risk factors		− 0.344		
I have been trained in important dietary guidelines including US Dietary Agency’s Guidelines for adults			0.614	
Unsaturated fatty acids are healthier than saturated fatty acids			0.412	
A BMI value > 18.5 is considered to be overweight among young adults	0.410			
A high caffeine intake can lead to increased heart rate and anxiety		0.407		
Folic acid supplements should be started in third trimester of pregnancy				0.349
Calcium supplementation is not important for patients with osteoporosis		0.335		
US dietary guidelines recommend less than 2 servings of dairy products per day for an adult				0.385
US dietary guidelines recommend between 6 and 11 servings of grain based products per day for an adult			0.423	
Patients with diabetes should be prescribed a diet with a low glycemic index to improve their blood sugar levels		0.3		
Meat products have the highest vitamin B-12 levels			0.368	
Vegetable oil has higher trans-fats than hydrogenated oils	0.705			
Women should have adequate exposure to sunlight to aid in vitamin D synthesis in their bodies	0.819			
Lower hemoglobin levels in blood may be due to poor levels of potassium in diet	0.849			
There are around 20 essential amino acids that are synthesized in human body and do not have to be taken from outside source				0.480
Bariatric surgery is a good treatment option for patients with extremely high BMI (> 40)	0.672			
It is important to refer most of my patients with obesity to nutritionists for expert advice		0.799		
I believe that a balanced nutrition is important for prevention of diseases including cardiovascular (atherosclerosis) and metabolic (diabetes mellitus) diseases		0.809		
Taking CME courses in nutrition in dietetics enhance my clinical practice, and management of patients		0.674		
Nutritionists are an important part of inter-disciplinary healthcare teams in hospitals		0.629		
It is important to calculate BMI and waist to hip ratio among my patients, to assess risk for cardiovascular diseases.		0.593		
Weight loss dietary regimens such as Atkin’s diet are poor for health	0.420			
I recommend specific diets (DASH, Mediterranean, Ketogenic etc.) in my clinical practice			0.475	
I am adequately trained to impart nutrition related counselling to patients			0.670	
I routinely prescribe green vegetable consumption to patients with kidney stones			0.375	
In my clinical practice, I routinely prescribe iron supplements to anemic mothers				0.550
I routinely perform nutrition related physical examinations to calculate BMI, waist to hip ratio and muscle mass among my patients			0.694	
I follow non-peer reviewed blogs to gain information on nutrition	0.500			
I am confident in prescribing lipid lowering agents such as statins for patients with cardiovascular risk factors and diseases				0.667
For patients with metabolic risk factors, I routinely calculate risk algorithms such as the Framingham Risk score or the Reynold Risk score				0.663
Serum triglyceride levels between 1.8 and 2.2 mmol/l (150 to 199 mg/dL) are considered in a very high range			0.473	
I routinely recommend high fibres diets to my patients, presenting with bowel problems	0.339			
For guidance related to nutrition, I use authentic sources such as text books, Medscape and up-to-date	0.484			

#### Data collection

Based on our preliminary analyses, we expected a two to four factor solution for this questionnaire, where the items presented wide communalities. We judged a sample size of 200 to be adequate for our study based on Comrey and Lee’s (1992) recommendation for sample size calculation for factor analysis. They recommended that a sample size of 50 to 100 is poor, 200 is fair, and above 300 is good [19, 20]. In addition, Mundform et al., recommended a sample size of 55 to 75 participants for scale items presenting with low communalities, two to four factor solution, variable to factor ratio of 12 and a good-level criterion (0.92) [19, 20].

Thereafter, we initiated the cross-sectional survey where a total of 200 dieticians, nutritionists and medical doctors were invited to participant in the survey, using convenient sampling method. Participants were recruited using an electronic survey developed using *Survey Monkey platform*. Professionals from several institutes and hospitals were contacted to participate in the survey during face to face meetings conducted at the Ministry of Health in Kuwait. Before participating in the survey, all the participants signed informed consent forms. Participation in the survey was voluntary, anonymous and the participants could leave the study at any time. Average time for completion of the questionnaire was around 20 min. Ethical approval for this study was provided by Ethical Review Board of Ministry of Health of Kuwait, Kuwait.

All data were analyzed using the SPSS v.25. Firstly, the data was subjected to dimension reduction using the Principal Component Analysis (PCA) and orthogonal rotation [18]. This process ascertained the dimensionality of the questionnaire by guiding the number of factors to retain and redundant items to be excluded. Before running the PCA, its suitability was assessed using the Kaiser-Meyer-Olkin (KMO) sampling adequacy statistic (> 0.60) and Bartlett’s test of sphericity. Number of factors to retain was based on three criteria; variance explained by each factor, Eigen value > 1 and the Cattell’s Scree Plot. Naming of each factor retained was done subjectively by analyzing the theme of most items included in the questionnaire. Suitability of each item was assessed using several criteria. For each item to be suitable for inclusion in the final scale, it was ensured that the KMO sampling adequacy value was > 0.6 for each item in the anti-image of the covariance matrix; communality value was ≥ 0.2 and the factor loading was ≥ 0.32.

Reliability analysis was done to evaluate the internal consistency of the overall scale, where a value ≥ 0.60 as considered to be acceptable [21]. Convergent validity was assessed using the Pearson’s correlation indices obtained using the inter-item correlations. Moreover, contribution to the overall Cronbach’s alpha value yielded by the scale was also assessed.

### Results

#### Demographics

A total of 173 (response rate = 86.93%) participants responded to the survey. There was a total of 92 (53.2%) nutritionists and 81 (46.8%) doctors/surgeons. Mean scores on individual items ranged from 1.99 (SD = 1.12) for item seven (BMI value > 18.5 is considered to be overweight) to 4.70 (SD = 0.50) for item 29 (referring patients with obesity to nutritionists).

### Factor validity

**Fig. 1:**
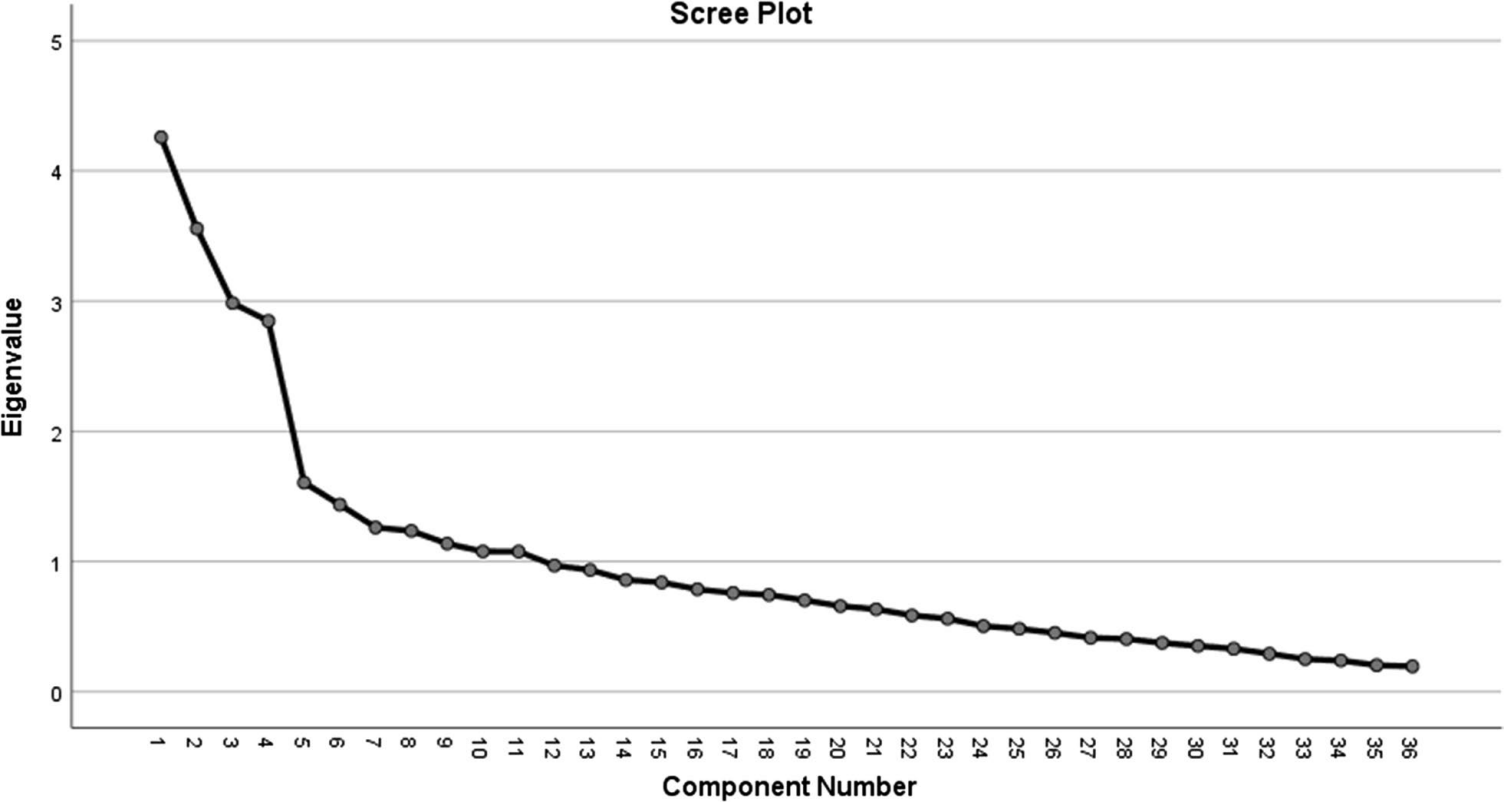
Scree plot for number of factors to retain

The KMO measure of sampling adequacy was found to be adequate at 0.71 (Bartlett’s test of sphericity P < 0.001). Each statement had demonstrated an adequate KMO value (≥ 0.60) in anti-image of correlation matrix. The factors to retain were assessed using several criteria. The criteria of Eigen value > 1 suggested that at least 11 factors should be retained. An assessment of the variance explained by the factor structure of the ATNQ showed that only four factors explained the highest proportion of variance. The Cattell’s scree plot suggested that only four factors be retained with the Eigen values ranging from 4.23 for factor to 2.85 for factor 4 (Fig. 1). These four factors cumulatively explained 37.9% of the variance in the factor structure of the ATNQ (Table 2).

**Table 2:** Reliability statistics

Item	Scale mean if item deleted	Scale variance if item deleted	Corrected item-total correlation	Squared multiple correlation	Cronbach’s alpha if Item deleted
Increased intake of fruits and vegetables is associated with good blood pressure control	115.6347	109.787	0.225	0.308	0.684
A traditional Mediterranean diet focuses on a reduction in total fat intake	116.5509	108.357	0.216	0.244	0.684
Research has shown strong evidence that Atkin’s low carbohydrate diet regime leads to a good cardiovascular health	117.1796	110.666	0.123	0.376	0.690
In comparison with nutrition, proper exercise is more important in reduction of cardiovascular risk factors	116.3713	114.994	− 0.084	0.351	0.708
I have been trained in important dietary guidelines including US Dietary Agency’s Guidelines for adults	116.5988	105.651	0.284	0.480	0.679
Unsaturated fatty acids are healthier than saturated fatty acids	115.5988	109.675	0.201	0.242	0.685
A BMI value > 18.5 is considered to be overweight among young adults	117.7305	109.367	0.153	0.309	0.689
A high caffeine intake can lead to increased heart rate and anxiety	115.4611	111.997	0.101	0.244	0.691
Folic acid supplements should be started in third trimester of pregnancy	117.6347	105.438	0.328	0.355	0.676
Calcium supplementation is not important for patients with osteoporosis	115.7425	114.192	− 0.044	0.366	0.701
US dietary guidelines recommend less than 2 servings of dairy products per day for an adult	117.0838	115.041	− 0.081	0.282	0.702
US dietary guidelines recommend between 6 to 11 servings of grain based products per day for an adult	116.2515	109.720	0.217	0.287	0.684
Patients with diabetes should be prescribed a diet with a low glycemic index to improve their blood sugar levels	115.7006	111.488	0.119	0.296	0.690
Meat products have the highest vitamin B-12 levels	115.8802	111.648	0.070	0.336	0.694
Vegetable oil has higher trans-fats than hydrogenated oils	116.9281	101.441	0.445	0.628	0.665
Women should have adequate exposure to sunlight to aid in vitamin D synthesis in their bodies	117.2814	101.276	0.430	0.674	0.666
Lower hemoglobin levels in blood may be due to poor levels of potassium in diet	116.9820	101.283	0.469	0.704	0.664
There are around 20 essential amino acids that are synthesized in human body and do not have to be taken from outside source	117.1078	113.012	0.011	0.346	0.697
Bariatric surgery is a good treatment option for patients with extremely high BMI (> 40)	117.4731	106.504	0.289	0.513	0.679
It is important to refer most of my patients with obesity to nutritionists for expert advice	115.0299	111.848	0.209	0.623	0.687
I believe that a balanced nutrition is important for prevention of diseases including cardiovascular (atherosclerosis) and metabolic (diabetes mellitus) diseases	115.0599	112.057	0.161	0.600	0.688
Taking CME courses in nutrition in dietetics enhance my clinical practice, and management of patients	115.2814	109.637	0.268	0.522	0.682
Nutritionists are an important part of inter-disciplinary healthcare teams in hospitals	115.0838	114.089	− 0.011	0.512	0.694
It is important to calculate BMI and waist to hip ratio among my patients, to assess risk for cardiovascular diseases	115.2335	108.698	0.330	0.427	0.679
Weight loss dietary regimens such as Atkin’s diet are poor for health	115.9880	108.446	0.239	0.431	0.683
I recommend specific diets (DASH, Mediterranean, Ketogenic etc.) in my clinical practice	116.0599	103.611	0.426	0.416	0.669
I am adequately trained to impart nutrition related counselling to patients	116.2515	109.021	0.167	0.547	0.688
I routinely prescribe green vegetable consumption to patients with kidney stones	116.8802	105.684	0.360	0.293	0.674
In my clinical practice, I routinely prescribe iron supplements to anemic mothers	116.4611	108.889	0.208	0.393	0.685
I routinely perform nutrition related physical examinations to calculate BMI, waist to hip ratio and muscle mass among my patients	116.3832	108.623	0.200	0.476	0.685
I follow non-peer reviewed blogs to gain information on nutrition	116.7365	101.689	0.464	0.431	0.664
I am confident in prescribing lipid lowering agents such as statins for patients with cardiovascular risk factors and diseases	116.3174	107.989	0.174	0.589	0.688
For patients with metabolic risk factors, I routinely calculate risk algorithms such as the Framingham Risk score or the Reynold Risk score	116.9042	110.870	0.096	0.499	0.693
Serum triglyceride levels between 1.8 and 2.2 mmol/l (150 to 199 mg/dL) are considered in a very high range	116.9760	109.698	0.169	0.298	0.687
I routinely recommend high fibers diets to my patients, presenting with bowel problems	117.1557	111.409	0.078	0.353	0.694
For guidance related to nutrition, I use authentic sources such as text books, Medscape and up-to-date	117.5749	109.704	0.171	0.267	0.687

Principle component analysis suggested that 16 items including item 4, 11, 18, 23, 28, 36, 39, 40, 41 and 44 cross-loaded strongly on two factors. And items 5, 8, 13, 19, 35 and 52 had lower factor loading < 0.30 (Additional file 1: Table S1). These items were removed from the final scale. Among the 36 remaining items (Table 2), the highest communality was shown by item 25 while communalities < 0.20 were exhibited by several statements including item 2, 12, 15, 20 and 50. However, these were not excluded and were kept for further assessment. All the items had an adequate communality ≥ 0.30. Highest factor loading was 0.85 demonstrated by statement 25, while the lowest communality was 0.34 by item 6. Item # 20 was removed from the overall scale at this point because it had shown a lower factor loading as well as communality.

#### Reliability statistics

The Cronbach’s alpha based on standardized items was 0.69 for the 36-item scale. Each item in the scale had at least one inter-item correlation ≥ 0.20, exhibiting appropriate convergent validity. Removing Item 6 (In comparison with nutrition, proper exercise is more important in reduction of cardiovascular risk factors) from the overall scale, improved the Cronbach’s alpha value to 0.71. In addition, an assessment of Cronbach’s alpha value of individual subscales revealed acceptable internal consistency. The first subscale named “Factual knowledge about nutrition” comprised of nine items. It yielded a Cronbach’s alpha value of 0.78. The second subscale “knowledge about nutrition in morbidities” comprised of seven items and yielded a Cronbach’s alpha value of 0.71. The third subscale “counselling of patients” comprised of 11 items and yielded a Conrbach’s alpha of 0.68. The fourth subscale comprising nine items yielded a Cronbach’s alpha value of 0.64 and was named, “Dietary programmes and supplementation”.

### Known group analysis

Total scores on the finalized scale were compared among physicians/surgeons and dieticians and nutritionists. The latter group scored higher on the scale (mean = 121.55, SD = 16.62) than the former (mean = 115.83, SD = 8.90). And this difference was found to be statistically significant.

### Discussion

This is the first study from Kuwait to report the development and validation of Attitude Toward Nutrition Counselling Questionnaire. It comprises of 36 items divided into four scales namely: (a) factual knowledge about nutrition; (b) knowledge about nutrition in morbidities; (c) counselling of patients and (d) dietary programmes and supplementation. This scale was found to have an acceptable reliability and validity.

During the development phase of the questionnaire, the original version comprised of 52 items. Several of these items were removed during the validation phase. For instance, item 4, 11, 18, 23, 28, 36, 39, 40, 41, 44 were removed because they exhibited strong factor loading on two different factors. While item 5, 8, 13, 19, 35 and 52 exhibited low factor loadings. These items weakened the construct validity of the questionnaire and therefore, were not included in the modified 35 item scale. Moreover, during the face validity period a few items were reworded. For instance, units for several metabolic parameters were changed from mg/dL to mmol/L that is more prevalent in medical practice. A few of the physicians also noted difficulty in understanding US dietary guidelines, however, these questions were kept in the questionnaire because no alternative guidelines were available for the Kuwaiti population.

## Limitations

This study has several strengths. An appropriate sample size was used for this study that comprised healthcare professionals across several specialties. It showed excellent internal consistency and validity among both the nutritionists and physicians and surgeons. This scale has several practical implications. It can be used to assess knowledge and attitudes of nutrition among doctors and assess areas/topics where training is required. It can thus, help to develop, tailor and tweak educational intervention packages for doctors. It can also be used to conduct pre and post assessment studies after delivery of an educational intervention regarding nutrition.

## Supplementary information

**Additional file 1: Table S1.** Rotated Component Matrix for the original 52 item scale.

## Data Availability

The dataset associated with this study, is available on request from the corresponding author.

## Abbreviations

PCA: Principal Component Analysis
KMO: Kaiser-Meyer-Olkin
ATNQ: Attitude Toward Nutrition Counselling Questionnaire
