# Supplementary material for: Development and validation of Attitude Toward Nutrition Counselling Questionnaire for use among Kuwaiti healthcare professionals
Source: BMC Res Notes. 2020 Feb 7;13:62. doi: 10.1186/s13104-020-4905-9 (PMC7006072; doi:10.1186/s13104-020-4905-9)
Supplement: Supplementary file 1 — Additional file 1: Table S1. Rotated Component Matrix for the original 52 item scale. [file 13104_2020_4905_MOESM1_ESM.docx]

**Supplementary Table 1: Rotated Component Matrix for the original 52 item scale**

| Item | **Statements** | **Component** | | | |
| --- | --- | --- | --- | --- | --- |
|  |  | **1** | **2** | **3** | **4** |
| 1 | Increased intake of fruits and vegetables is associated with good blood pressure control. |  |  | 0.503 |  |
| 2 | A traditional Mediterranean diet focuses on a reduction in total fat intake. |  |  |  | 0.388 |
| 3 | Research has shown strong evidence that Atkin’s low carbohydrate diet regime leads to a good cardiovascular health. |  |  |  | 0.463 |
| 4 | High intake of trans fatty acids increases low density lipoprotein (LDL) as well as high density lipoprotein (HDL) levels. | 0.368 |  |  | 0.426 |
| 5 | High sodium intake is associated with a higher risk of hypertension. |  |  |  |  |
| 6 | In comparison with nutrition, proper exercise is more important in reduction of cardiovascular risk factors. |  |  |  | 0.33 |
| 7 | I have been trained in important dietary guidelines including US Dietary Agency’s Guidelines for adults. |  |  | 0.606 |  |
| 8 | US dietary guidelines recommend more than 7 servings of proteins per day for an adult. |  |  |  |  |
| 9 | Unsaturated fatty acids are healthier than saturated fatty acids. |  |  | 0.383 |  |
| 10 | A BMI value > 18.5 is considered to be overweight among young adults. | 0.362 |  |  |  |
| 11 | US dietary guidelines recommend more than 7 servings of fruits per day for an adult. |  | -0.332 |  | 0.343 |
| 12 | A high caffeine intake can lead to increased heart rate and anxiety. |  | 0.393 |  |  |
| 13 | Zinc is a dietary trace element that plays a role in cell division, and maturation. |  |  |  |  |
| 14 | Folic acid supplements should be started in third trimester of pregnancy. |  |  |  | 0.383 |
| 15 | Calcium supplementation is not important for patients with osteoporosis. |  | -0.327 |  |  |
| 16 | US dietary guidelines recommend less than 2 servings of dairy products per day for an adult. |  |  |  | 0.36 |
| 17 | US dietary guidelines recommend between 6 to 11 servings of grain based products per day for an adult. |  |  | 0.393 |  |
| 18 | Statin drugs are important prescription medicines for prevention of cardiovascular diseases such as stroke and heart attack. | -0.373 | 0.324 |  | 0.348 |
| 19 | Diet with lower pro-inflammatory scores lead to lower risk of cancers. |  |  |  |  |
| 20 | Patients with diabetes should be prescribed a diet with a low glycemic index to improve their blood sugar levels. |  | 0.364 |  |  |
| 21 | Meat products have the highest vitamin B-12 levels. |  |  | 0.371 |  |
| 22 | Vegetable oil has higher trans-fats than hydrogenated oils. | 0.704 |  |  |  |
| 23 | Proteins contain higher number of calories than carbohydrates and fats. |  | -0.485 |  | 0.345 |
| 24 | Women should have adequate exposure to sunlight to aid in vitamin D synthesis in their bodies. | -0.825 |  |  |  |
| 25 | Lower hemoglobin levels in blood may be due to poor levels of potassium in diet. | 0.837 |  |  |  |
| 26 | There are around 20 essential amino acids that are synthesized in human body and do not have to be taken from outside source. |  |  |  | 0.465 |
| 27 | Bariatric surgery is a good treatment option for patients with extremely high BMI (> 40). | -0.654 |  |  |  |
| 28 | It is important to counsel patients regarding proper nutrition. | -0.392 | 0.4 |  |  |
| 29 | It is important to refer most of my patients with obesity to nutritionists for expert advice. |  | 0.764 |  |  |
| 30 | I believe that a balanced nutrition is important for prevention of diseases including cardiovascular (atherosclerosis) and metabolic (diabetes mellitus) diseases. |  | 0.774 |  |  |
| 31 | Taking CME courses in nutrition in dietetics enhance my clinical practice, and management of patients. |  | 0.622 |  |  |
| 32 | Nutritionists are an important part of inter-disciplinary healthcare teams in hospitals. |  | 0.633 |  |  |
| 33 | It is important to calculate BMI and waist to hip ratio among my patients, to assess risk for cardiovascular diseases. |  | 0.576 |  |  |
| 34 | Weight loss dietary regimens such as Atkin’s diet are poor for health. | 0.451 |  |  |  |
| 35 | Dietary life styles such as Mediterranean diets are good for patients with Diabetes mellitus and atherosclerosis. |  |  |  |  |
| 36 | Nutrition related education should be made a part of undergraduate syllabus in medicine. | -0.323 | 0.423 |  |  |
| 37 | I recommend specific diets (DASH, Mediterranean, Ketogenic etc) in my clinical practice. |  |  | 0.437 |  |
| 38 | I am adequately trained to impart nutrition related counselling to patients. |  |  | 0.714 |  |
| 39 | Proper nutrition is important in reduction of cardiovascular risk factors. | -0.355 | 0.523 |  |  |
| 40 | I calculate my patient’s daily energy requirements and chart out nutritional requirements. | 0.417 |  | 0.726 |  |
| 41 | I feel adequately trained in providing parenteral nutritional therapy to sick patients. | 0.549 |  | 0.494 |  |
| 42 | I routinely prescribe green vegetable consumption to patients with kidney stones. |  |  | 0.365 |  |
| 43 | In my clinical practice, I routinely prescribe iron supplements to anemic mothers. |  |  |  | 0.53 |
| 44 | I prescribe micronutrient supplements to patients with different diseases such as scurvy, anemia and hair fall etc. |  |  | 0.344 | 0.393 |
| 45 | I routinely perform nutrition related physical examinations to calculate BMI, waist to hip ratio and muscle mass among my patients. |  |  | 0.68 |  |
| 46 | I follow non-peer reviewed blogs to gain information on nutrition. | 0.475 |  |  |  |
| 47 | I am confident in prescribing lipid lowering agents such as statins for patients with cardiovascular risk factors and diseases. |  |  |  | 0.637 |
| 48 | For patients with metabolic risk factors, I routinely calculate risk algorithms such as the Framingham Risk score or the Reynold Risk score. |  |  |  | 0.564 |
| 49 | Serum triglyceride levels between 1.8 to 2.2 mmol/l (150 to 199 mg/dL) are considered in a very high range. |  |  | 0.452 |  |
| 50 | I routinely recommend high fibers diets to my patients, presenting with bowel problems. | -0.34 |  |  |  |
| 51 | For guidance related to nutrition, I use authentic sources such as text books, Medscape and up-to-date. | -0.445 |  |  |  |
| 52 | I counsel patients with chronic kidney disease, on reducing salt and protein intake. |  |  |  |  |
